# Interaction Between Apolipoprotein M Gene Single-Nucleotide Polymorphisms and Obesity and its Effect on Type 2 Diabetes Mellitus Susceptibility

**DOI:** 10.1038/s41598-020-64467-6

**Published:** 2020-05-12

**Authors:** Dan Liu, Jian-Min Pan, Xiang Pei, Jun-Sen Li

**Affiliations:** 1 Department of Endocrinology, The third people’s Hospital of Hainan Province, Sanya, China; 2 Department of general surgery, The third people’s Hospital of Hainan Province, Sanya, China

**Keywords:** Genetic association study, Diabetes

## Abstract

This study investigated the correlation of four single nucleotide polymorphisms (SNPs) in Apolipoprotein M (ApoM) with the risk of type 2 diabetes mellitus (T2DM) and effects of the interactions of this gene and obesity. The effects of SNP and obesity interaction on T2DM was examined by generalized multifactor dimensionality reduction (GMDR) combined with the logistic regression model. T2DM patient-control haplotype was analyzed *in silico* using the haplotype analysis algorithm SHEsis. The rs805296-C allele or 724-del allele indicted high risk of T2DM. The incidence of T2DM in individuals with rs805296-C allele polymorphism (TC + CC) was higher than those without (TT), adjusted OR (95%CI) = 1.29 (1.10–1.66) (*p* < 0.001). Moreover, the individuals with 724-delallele have a higher risk of T2DM compared to those with 724-ins variants, adjusted OR (95%CI) = 1.66 (1.40–2.06), *p* < 0.001. GMDR analysis suggested that the interaction model composed of the two factors, rs805296 and obesity, was the best model with statistical significance (P value from sign test [*P*_sign_]=0.0107). The T2DM risk in obese individuals having TC or CC genotype was higher than non-obese individuals with TT genotype (OR = 2.38, 95% CI = 1.58–3.53). Haplotype analysis suggests that rs805297-C and rs9404941-C alleles haplotype indicate high risk of T2DM, OR (95%CI) = 1.62 (1.29–2.16), *p* < 0.001. Our results suggested that rs805296 and 724-del minor allele of ApoM gene, interaction of rs805296 and obesity, rs805297-C and rs9404941-C alleles haplotype were indicators of high T2DM risk.

## Introduction

Among adults in China, the estimated overall prevalence ofdiabetes was 10.9%, and that for prediabetes was 35.7%. Thus, the prevalence of type 2 diabetes mellitus (T2DM) in China is the highest in the world^1^, and the number of patients with T2DM will be about 438 million in 2030^2,3^. Unfortunately, the incidence of T2DM will continue to increase in the next decades in many countries, including China, due to longevity of human life and obesity^4^. The development and progression of T2DM are believed to be closely correlated with the interaction of multiple susceptibility genes and gene interactions with the environment^5–7^.

Human apolipoprotein M gene is structurally conserved across species and located at the human chromosome 6p21.33^8,9^. ApoM is reported to be highly expressed in liver and kidneys, but weakly expressed in other human tissues^10^. Previous studies reported associations between ApoM gene variations and human diseases, including CAD and T2DM; however, these observations remain controversial^11–15^. In Chinese populations, Xu *et al*.^11^ showed the ApoM rs9404941 (T-855C) polymorphism predicts a high incidence of CAD. Furthermore, ApoM rs805296 (T-778C) polymorphism was closely related with the incidence of either type 1 or type 2 diabetes^12,13^. However, the association of Apo Mgene polymorphism in rs805296 (T-778C) and the risk of T2DM was found in another independent study based on Southern Chinese population^14^. Emerging evidence showed that genetic and environmental determinant factors were co-contributors for the initiation and progression of T2DM. It was reported that genetic background is a key modulator for the human reactions to environmental determinant factors. The effects of obesity on the incidence of T2DM have been extensively studied in different populations^16,17^. However, the impact of gene- environment interaction between ApoM gene and obesity onT2DM risk was not studied in Chinese population until now. Therefore, we investigated the associations of four ApoM gene SNPs and the risk of T2DM. Furthermore, we studied the interaction of ApoM gene and obesity on risk of T2DM in this case-control study.

## Materials and methods

### Subjects

A total of 681 patients diagnosed with T2DM receivingtreatment between Mar 2011 and Dec 2015 at the third people’s Hospital of Hainan Province were enrolled in this study. Patients with diseases including thyroid, hematologic neoplastic, cardiac, hepatic, or non-diabetic kidney disease were excluded in this study. Healthy controls were recruited from patients without T2DM, with a nearly 1:1 matched (age and sex). Selected controls were in good health, with normal fasting blood glucose and glucose intolerance but without significant medical history in previous. Signed informed consent was provided by all the recruited participants. The protocol of this study was approved by the Ethics Committee of the third people’s Hospital of Hainan Province. All research methods in this study were carried out bythe approved guidelines.

### Body measurements

General clinicopathological information of enrolled participants were recorded by trained staff. Body weight in kilograms divided by the square of the height in meters was used to measure body mass index (BMI). Individuals with cigarette smoking history for one year or above and smoke at least once per day were classified as cigarette smokers. Alcohol amount consumed was the sum alcohol consumed per week from all kinds of wine. After 8 or more hours of fasting, blood samples were obtainedfrom the individuals in the next morning. Samples were stored at –80 °C until use. Oxidase enzymatic method was used to measure plasma glucose concentration. High-density lipoprotein (HDL)- cholesterol and triglyceride (TG) concentrations were measured using a Hitachi biochemistry.

### Genomic DNA extraction and genotyping

dbSNP algorithm (http://www.ncbi.nlm.nih.gov/projects/SNP)was used to select the SNPs to be investigated (criteria: MAF > 5% in the dbSNP database). Four SNPs (rs805296,−724 ins/del, rs805297 and rs9404941) were selected for further investigations in this study. The genomic DNA was extracted from the collected patients or healthy individuals blood samples using Genomic DNA extraction kit (Roche, USA) and stored at −80 °C. The genotype of the selected SNPs in the samples was analyzed by Restriction Fragment Length Polymorphism (RFLP) on the basis of polymerase Chain Reaction (PCR). The primer sequences and descriptions of 4 SNPs are shown in Table 1. The PCR reaction system included: Taq DNA polymerase, dNTPs, PCR buffer, and MgCl_2_. The measurement of PCR detection reagent is as follows: less than 0.1 μg genomic DNA template, 12.5 μl 2 × Taq PCR Mastermix, 10 μmol of each primer and add ddH_2_O to a final reaction volume of 25 μl. PCR was carried out at an Applied Biosystems PCR equipment using the following procedures: 1 cycle of 94 °C denaturation for 3 min, 30 cycles of 95 °C denaturation for 30 s, 60 °C annealing for 30 s, and 72 °C extension for 30 s. The resulted products were sequenced using an automatic sequencer (Model 3730, BGI, Shanghai, China).

**Table 1:** The detailed introduction forfour SNPs within ApoM gene.

SNP	rs number	Chromosome	Functional Consequence	Probe sequence
C-1065A	rs805297	6:31654829	Intron variant, upstream variant 2KB	F: 5′- GCTTTGCAAACATTACTATTCAT-3′ R: 5′- ATTGGCAAATCATCAATCTTATA-3′
T-778C	rs805296	6:31655116	Intron variant, upstream variant 2KB	F: 5′-ATAGCAGTTAGGGGTTGGTGG-3′ R: 5′-CTCTTCCGGATGCAACCACT-3′
T-855C	rs9404941	6:31655039	Intron variant, upstream variant 2KB	F: 5′-ATAGCAGTTAGGGGTTGGTGG-3′ R: 5′-CTCTTCCGGATGCAACCACT-3′
C-724del	—	Promoter region	Missing	F: 5′-AGTCACTTGGT GCTATCC-3′ R: 5′-GTTGGTGTCAGGCAGAAT-3′

### Diagnostic criteria

Individuals with a fasting glucose ≥ 126 mg/dl (7.0 mmol/l) or having undergone hypoglycemic therapy were diagnosed as diabetics. Individuals with a fasting glucose ≥ 126 mg/dl (7.0 mmol/l), or blood glucoselevels2 h postprandial ≥ 200 mg/dl (11.0 mmol/l), or having undergone hypoglycemic therapy in the interim were classified into T2DM group^18^. Individuals with a BMI ≥ 28 kg/m^2^ were classified as obese^19^.

### Statistical analysis

All data analysis was performedon SPSS 22.0 software (Chicago, IL). Mean and standard deviation (SD) were measured for continuous variables and the differences were analyzed using Students’ t- test; percentages were measured for categorical variables and the differences were analyzed using chi-square test. The genotype distribution differences among individuals with T2DM and healthy controls were analyzed usingChi-square test. In silico analysis algorithm SHEsis was used toanalyze T2DM patient-control haplotype (http://analysis.bio-x.cn/myAnalysis.php). Generalized multifactor dimensionality reduction (GMDR) was performed to investigate all the interactions. Effects of SNPs and obesity interaction on the risk of T2DM were measured by logistic regression model. The collected clinicopathologicalinformation was used to adjust odds.

## Results

A total of 1371 participants, consisting of 681 in the T2DM group and 690 as healthy controls, were enrolled (606 males and 765 females) with a mean age at 61.1 ± 13.8. The general characteristics of these enrolled T2DM patients and healthy controls are shown in Table 2. We observed a significant distribution difference between T2DM patients andhealthy controls in BMI, TG, TC, and HDL. However, no close association was observed for males, smoking, alcohol consumption, and mean age between T2DM patients and controls.

**Table 2:** General characteristics of the enrolled T2DM patients and controls.

Variables	T2DM patients (n = 681)	Controls (n = 690)	*P-*values
Age	60.7 ± 14.3	61.4 ± 14.6	0.370
Males (N)	310(45.5%)	296(42.9%)	0.328
Smoke (N)	173 (25.4%)	164 (23.8%)	0.482
Alcohol drinking (N)	144(21.1%)	136(19.7%)	0.510
BMI(kg/m^2^)	24.6 ± 6.4	23.2 ± 6.7	<0.001
TG (mmol/L)	2.1 ± 0.67	1.8 ± 0.70	<0.001
TC (mmol/L)	5.3 ± 1.2	4.7 ± 1.1	<0.001
HDL (mmol/L)	1.21 ± 0.33	1.32 ± 0.27	<0.001

Note: median and inter quartile for TG; means ± standard deviation for age, BMI, TC, HDL-C; TC, total cholesterol; HDL, high density lipoprotein; TG, triglyceride.

Genotype distribution was analyzed using the Hardy–Weinberg equilibrium. The frequenciesof C allele of **rs805296**and **724- del**were higher in individuals with T2DM compared to healthy controls (30.4% of T2DM patients and 22.2% of controls, *p* < 0.001 for C allele of **rs805296;** 28.9% of T2DM patients and 21.2% of controls, *p* < 0.001 for **724-del**) (Table 3). Logistic regression model revealedthe incidence of T2DM in individuals with **rs805296**-C allele or **724-del** allelewas higher compared to those with TT variants or **724-ins** variants respectively (Table 3). However, no significant correlationswere found when the association of C-1065Ars805297 and T-855Crs9404941 with T2DM risk (Table 3) were analyzed.

**Table 3:** Genotype and allele frequencies of four SNPs in T2DM patientsand controls.

SNPs	Genotypes and Alleles	Frequencies n (%)		OR(95%CI)^*^	*P*-values	*HWE test*
		T2DM patients (n = 681)	Controls(n = 690)			
**T-778C rs805296**	TT	339 (49.8)	422 (61.2)	1.00	<0.001	0.369
	TC	270 (39.6)	230 (33.3)	1.24 (1.02–1.56)		
	CC	72 (10.6)	38 (5.5)	1.83 (1.24–2.51)		
	TC + CC	342 (50.2)	268 (38.8)	1.29 (1.10–1.66)	<0.001	
	T	948 (69.6)	1074 (77.8)		<0.001	
	C	414(30.4)	306 (22.2)			
C-1065A rs805297	GG	359 (52.7)	396 (57.4)	1.00	0.119	0.147
	GT	256 (37.6)	244 (35.4)	1.06 (0.95–1.37)		
	TT	66 (9.7)	50 (7.2)	1.10 (0.86–1.61)		
	GT + TT	322 (47.3)	294 (42.6)	1.07 (0.92–1.43)	0.082	
	G	974 (71.5)	1036 (75.1)		0.035	
	T	388 (28.5)	344 (24.9)			
**−724 ins/del**	Ins/ ins	346 (50.8)	431 (62.5)	1.00	<0.001	0.631
	Ins/ del	276 (40.5)	226 (32.8)	1.61 (1.38–1.89)		
	Del/ del	59 (8.7)	33 (4.8)	2.03(1.62–2.83)		
	Ins/ del+Del/ del	335 (49.2)	259 (37.5)	1.66 (1.40–2.06)	<0.001	
	Ins	968 (71.1)	1088 (78.8)		<0.001	
	Del	394(28.9)	292 (21.2)			
T-855C rs9404941	TT	364 (53.4)	402 (58.3)	1.00	0.188	0.247
	TC	263 (38.6)	242 (35.1)	1.08(0.91–1.36)		
	CC	54 (7.9)	46 (6.7)	1.04 (0.82–1.53)		
	TC + CC	317 (46.5)	288 (41.7)	1.07 (0.89–1.39)	0.073	
	T	991 (72.8)	1046(75.8)		0.069	
	C	371 (27.2)	334(24.2)			

^*^Adjusted for gender, age, smoke and alcohol consumption status, high fat diet, low fiber diet, TC and HDL.

After covariates adjustment, GMDR analysis was performed to analyze the correlation of ApoM gene and obesity interaction with the risk of T2DM (Table 4). The results revealed that the interaction model composed of the two factors, **rs805296** and obesity, which was the best model with statistical significance (P value from sign test [*P*_sign_]=0.0107). Meanwhile, the cross-validation consistency and testing accuracy for this two- locus were 10/10 and 62.17%, respectively. After adjustment for the collected clinicopathological information, we found the incidence of T2DM in individuals with TC or CC genotype and high BMI was higher than those with TT genotype and normal BMI(Table 5).

**Table 4:** GMDR to predict the gene- obesity interaction models.

Locus no.	Combinations	Cross-validation consistency	Testing accuracy	*P- values* ^*^
2	**rs805296**Obesity	10/10	0.6217	0.0107
3	**rs805296–724 ins/del**Obesity	9/10	0.5577	0.1719
4	**rs805296-724 ins/del**rs805297Obesity	8/10	0.5590	0.0547
5	**rs805296-724 ins/del**rs805297rs9404941 Obesity	7/10	0.4958	0.3770

^*^Adjusted for gender, age, smoke and alcohol consumption status, high fat diet, low fiber diet, TC and HDL.

**Table 5:** Logistic regression to analyze the interactions betweenrs 805296 and obesity.

rs805296	Obesity	OR (95% CI) ^*^	*P*-values
TT	No	1.00	—
TC or CC	No	1.20 (1.06–1.48)	0.030
TT	Yes	1.49 (1.10–1.89)	0.001
TC or CC	Yes	2.38 (1.58–3.53)	<0.001

^*^Adjusted for gender, age, smoke and alcohol consumption status, high fat diet, low fiber diet, TC and HDL.

D’ values of4 ApoM gene SNPs were analyzed using Pairwise LD statistics. The results presented in Table 6 showed that value calculated for rs9404941 and rs805297was 0.817. Therefore, analysis for rs9404941 and rs805297 was performed within silico haplotype analysis softwareSHEsis. As a result, the frequency of T-T haplotype was the highest in both populations (47.01% for individuals with T2DM, 54.67% for healthy controls). Also, our results demonstrated that rs805297-C and rs9404941-C alleles were indicators for a high T2DM risk (Table 7).

**Table 6:** D’ values among SNPs in ApoM gene using linkage disequilibrium test.

SNPs	D’ values		
	rs805296	rs9404941	C724del
rs805297	0.268	**0.817**	0.411
rs805296	—	0.319	0.197
rs9404941			0.334

**Table 7:** ApoM gene and T2DM risk association measured by haplotype analysis.

*Haplotypes*		Frequencies		OR (95%CI)	*P*-values^*^
		T2DM patients	Controls		
T	T	0.4701	0.5467	1.00	–
C	T	0.2167	0.2131	1.12 (0.80–1.66)	0.628
T	C	0.2015	0.1971	1.26 (0.85–1.75)	0.435
C	C	0.1117	0.0431	1.62 (1.29–2.16)	<0.001

*Adjusted for gender, age, smoking and BMI.

## Discussion

Our research indicated that T2DM risk was positively correlated with **rs805296**-C or **724-del** allele but did not have any association with the other two SNPs. Although different studies have identified several potential candidate genes associated with the risk of T2DM risk factors, including SNPs polymorphism within ApoM gene^12–15^. However, the conclusions drawn from these reports regarding the relationship between ApoM SNPs and incidence of T2DMare inconsistent. For example, two Chinese studies indicated that ApoM rs805296 (T-778C) was an indicator forthe risk of both type 1 and type 2 diabetes^12,13^. However, nocorrelationbetween this specific gene polymorphism and the risk of T2DM was found in an independent study^14^. Zhang *et al*.^20^ analyzed the associations between four SNPs investigated in this study and the risk of T2DM in a total of 335 eastern Han Chinese participants. The resultsillustrated that C-724del polymorphism predicts a high risk of T2DM but rs805296 (T-778C) polymorphism did not correlate with the risk of T2DM. Moreover, the genotype frequency and distribution difference of rs805297 (C-1065A) and rs9404941 (T-855C) inindividuals with T2DM and healthy controls was not significant. Xu *et al*^11^. suggested that ApoM rs9404941 (T-855C) predicts highincidenceof CAD^8^. But another study^21^ indicated that rs805297 SNP in ApoM gene predicts high risk of dyslipidemia but did not have any influence on the incidence of CAD. A previous study^22^ also suggested that plasma ApoMexpressionwasupregulated in individuals with hyperlipidemia butdownregulated in individuals with T2DM compared with that in healthy controls. In addition, previous studiesimplied that ApoM expression was inversely correlated withglycemia^23^. Moreover, overexpression of ApoM in Goto-Kakizaki rats enhanced the effects of insulin^24^, indicating that ApoMhas the potential to be used a therapeutic target for T2DM.

It was reported that genetic background is a key modulator for the human reactions to environmental risk factors. The importance of environmental risk factors including obesity in the pathogenesis of T2DM has been widely recognized^15,16^. However, no study was performed to analyze the gene-environment interaction, especially the interaction ofApoM and obesity on the incidence of T2DM. Therefore, GMDR model was used to analyze the interaction of ApoMand obesity onthe incidence of T2DM in Chinese population;it revealed that the interaction of **rs805296** and obesity was significant. We also found that TC or CC genotype and high BMI increased the incidence of T2DM in comparisonto TT genotype and normal BMI. In this study, we showed a string chain reaction between rs805297 and rs9404941 since the D’ value was above 0.8. We also found that individuals with rs805297-C and rs9404941-C allelestend to have high risk of T2DM using haplotype analysis.

There were several shortcomings in this study. Firstly, limited numbers ofApoM SNPswere investigated and may result in the genetic information of ApoM was not sufficientlyfactored into the analysis. Secondly, the numbers of enrolled individuals were small and therefore a clinical study with a large sample size should be performed. Thirdly, IR level was not measured.

In conclusion, **rs805296** and **724-del** minor allele of ApoM gene, **rs805296**-obesity interaction, and the alleles rs805297-C and rs9404941-C were risk factors for the development and progression of T2DM.

### Ethics approval and consent to participate

This study has been approvedby ethics committee of the third people’s Hospital of Hainan Province.
